# Intermolecular Failure of L-type Ca^2+^ Channel and Ryanodine Receptor Signaling in Hypertrophy

**DOI:** 10.1371/journal.pbio.0050021

**Published:** 2007-01-09

**Authors:** Ming Xu, Peng Zhou, Shi-Ming Xu, Yin Liu, Xinheng Feng, Shu-Hua Bai, Yan Bai, Xue-Mei Hao, Qide Han, Youyi Zhang, Shi-Qiang Wang

**Affiliations:** State Key Lab of Biomembrane and Membrane Biotechnology, Ministry of Education Key Lab of Molecular Cardiovascular Sciences and Institute of Vascular Medicine, Third Hospital, College of Life Sciences, Peking University, Beijing, China; Babraham Institute, United Kingdom

## Abstract

Pressure overload–induced hypertrophy is a key step leading to heart failure. The Ca^2+^-induced Ca^2+^ release (CICR) process that governs cardiac contractility is defective in hypertrophy/heart failure, but the molecular mechanisms remain elusive. To examine the intermolecular aspects of CICR during hypertrophy, we utilized loose-patch confocal imaging to visualize the signaling between a single L-type Ca^2+^ channel (LCC) and ryanodine receptors (RyRs) in aortic stenosis rat models of compensated (CHT) and decompensated (DHT) hypertrophy. We found that the LCC-RyR intermolecular coupling showed a 49% prolongation in coupling latency, a 47% decrease in chance of hit, and a 72% increase in chance of miss in DHT, demonstrating a state of “intermolecular failure.” Unexpectedly, these modifications also occurred robustly in CHT due at least partially to decreased expression of junctophilin, indicating that intermolecular failure occurs prior to cellular manifestations. As a result, cell-wide Ca^2+^ release, visualized as “Ca^2+^ spikes,” became desynchronized, which contrasted sharply with unaltered spike integrals and whole-cell Ca^2+^ transients in CHT. These data suggested that, within a certain limit, termed the “stability margin,” mild intermolecular failure does not damage the cellular integrity of excitation-contraction coupling. Only when the modification steps beyond the stability margin does global failure occur. The discovery of “hidden” intermolecular failure in CHT has important clinical implications.

## Author Summary

High blood pressure induces hypertrophy, a thickening of the cardiac muscle that eventually leads to heart failure, a leading cause of morbidity and mortality. The contractile power of the heart depends in part on signaling between calcium channels on the cell membrane (L-type Ca^2+^ channels) and calcium release channels on a specialized calcium-regulating organelle called the sarcoplasmic reticulum. This signaling process is defective in heart failure. We have found that the signaling efficiency between a single L-type channel and its controlled Ca^2+^ release channels decreases during the transition from hypertrophy to heart failure. Moreover, we find unexpectedly that the signaling failure between channels occurs even before any obvious defect in the cardiac cell's ability to contract is seen. In normal cells, the timing between calcium influx and release is rapid; but in hypertrophy before heart failure manifests, there is a delay in this signaling process. In seeking the underlying mechanisms of this intermolecular failure, we find that a protein known as junctophilin, which anchors the sarcoplasmic reticulum to the cell membrane system, is expressed at a lower level. These results reveal early molecular events associated with the progression of hypertrophy, and may provide new insights for developing methods of early diagnosis and treatment to prevent heart failure.

## Abstract 3

The authors show that although whole-cell coupling of L-type calcium channels and ryanodine receptor current activation remains intact during compensated hypertrophy (before heart failure manifests), intermolecular coupling at a molecular level is already slipping.

## Introduction

In response to pressure overload, the heart produces an adaptive response in the form of cardiac hypertrophy to maintain adequate cardiac output and tissue perfusion [1–3]. In the early stage of hypertrophy, cardiac contractile dysfunction is not present, and the ventricle is hemodynamically compensated. When the pressure stimuli are persistent, the heart usually undergoes functional deterioration, eventually leading to heart failure [3,4]. In the failure stage, the heart becomes incapable of generating sufficient pumping power. To prevent the pathogenesis of heart failure, one strategy has been to stop or postpone the transition of hypertrophy from the compensated stage toward the decompensated stage [4]. Therefore, understanding the cellular and molecular mechanisms involved in cardiac hypertrophy is important for developing clinical therapies against heart failure.

At the cellular level, the contractile power during excitation-contraction coupling (E-C coupling) is governed by a mechanism known as Ca^2+^-induced Ca^2+^ release (CICR) [5,6]. In this process, Ca^2+^ influx through L-type Ca^2+^ channels (LCCs) on the cell surface membrane (including T-tubules) activates ryanodine receptor (RyR) Ca^2+^ release from the sarcoplasmic reticulum (SR) to generate cell-wide Ca^2+^ transients [7–9]. Besides LCCs and RyRs, Ca^2+^ cycling proteins, e.g., SR Ca^2+^ pumps (SERCA), Na^+^-Ca^2+^ exchangers, and their regulatory mechanisms, are also important in determining the amplitude and kinetics of Ca^2+^ transients [8]. All these mechanisms have been studied in a wide variety of hypertrophy and heart failure models [8,10–14]. Most studies support the idea that the LCC activity does not change much during hypertrophy and heart failure [11]. However, the Ca^2+^ transients triggered by comparable LCC currents are decreased in amplitude and/or slowed in kinetics in most models of decompensated hypertrophy (DHT) and heart failure [11,13]. These studies lead to the notion that the Ca^2+^ influx through LCCs becomes less effective in triggering RyR Ca^2+^ release [13]. Yet the molecular details underlying defective E-C coupling remain unknown. On the other hand, studies on compensated hypertrophy (CHT), a stage prior to DHT, show that the cellular aspects of E-C coupling still appear to be normal or even slightly enhanced [15]. It is thus intriguing to know whether and when the intermolecular process of CICR is modified and how the modification eventually leads to cellular failure in E-C coupling.

During past years, we have developed a local Ca^2+^ imaging protocol in conjunction with a loose-seal patch clamp technique to investigate LCC-RyR intermolecular coupling [9,16]. In the present study, we utilized this technique and an aortic stenosis model to test the hypothesis that the intermolecular coupling between an LCC and RyRs undergoes a progressive modification during the development of hypertrophy. Our results showed that hypertrophy resulted in an increase in LCC-RyR coupling latency and a decrease in intermolecular signaling efficiency, which started at the early, compensated stage when cellular E-C coupling appeared normal. Our findings provided intermolecular insights into the remodeling of Ca^2+^ signaling during the pathogenesis leading to heart failure.

## Results

**Figure 1:**
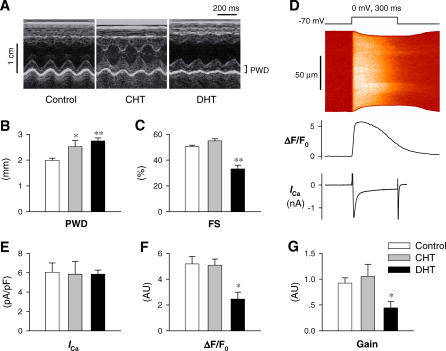
Functional Characterization of the Aortic Stenosis Model (A) Representative echocardiograms used to measure the ventricle wall thickness during cardiac cycles. (B) and (C) Posterior wall thickness (PW_d_) (B) and fractional shortening (FS) (C) measured by echocardiography were compared among the control (*n* = 13), CHT (*n* = 9), and DHT (*n* = 8) groups. A single asterisk (*) indicates *p* < 0.05, and double asterisks (**) indicate *p* < 0.01 compared with the control. (D) Whole-cell patch clamp combined with line-scan confocal imaging was used to record simultaneously LCC Ca^2+^ currents (*I*_Ca_, bottom) and Ca^2+^ transients in ventricular myocytes from the control, CHT, and DHT groups. Only a sample from CHT is shown to avoid redundancy. The fluo-4 fluorescence *(F)* was normalized to background fluorescence before depolarization (*F*_0_). Ca^2+^ transient parameters were determined based on the spatial average of *F/F*_0_ (middle panel). (E) and (F) The amplitude of *I*_Ca_ normalized to cell capacitance (E) and the peak amplitude of the Ca^2+^ transient (F) were compared between groups. AU, arbitrary units; pA/pF, pico ampere per pico faraday. (G) The gain of E-C coupling was calculated as the peak *F/F*_0_ per unit *I*_Ca_ density.

To elucidate the microscopic modification of E-C coupling during hypertrophy, we created pressure-overload hypertrophy models induced by aortic stenosis [17]. About 7–11 wk after aorta banding, hemodynamic and echocardiographic measurements identified the status of CHT by increased left ventricle (LV) wall thickness and normal contractile indices, and the status of DHT by the onset of mild depression of contractile indices in addition to thickened LV walls (Figure 1A, 1B, and 1C; Table S1). To characterize the cellular aspects of E-C coupling, we combined a whole-cell patch clamp technique and confocal line-scan imaging to record simultaneously LCC Ca^2+^ current (*I*_Ca_) and intracellular Ca^2+^ transients when the cell membrane was depolarized to 0 mV (Figure 1D). Cell capacitance (Figure S1A) and contraction (Figure S1B) were also measured. In DHT, despite the unchanged *I*_Ca_ density and kinetics (Figure 1E and Figure S1C and S1D), both the amplitude of Ca^2+^ transients and cell contraction decreased significantly (Figure 1F and Figure S1B). As a result, the gain of E-C coupling was significantly lower than that of the control (Figure 1G). By contrast, neither the amplitude of Ca^2+^ transients nor the gain of E-C coupling was altered in CHT, indicating that the hypertrophy-associated E-C coupling deficiency occurs only in the late, decompensated stage, but not in the early, compensated stage.

### LCC-RyR Coupling during Hypertrophy

**Figure 2:**
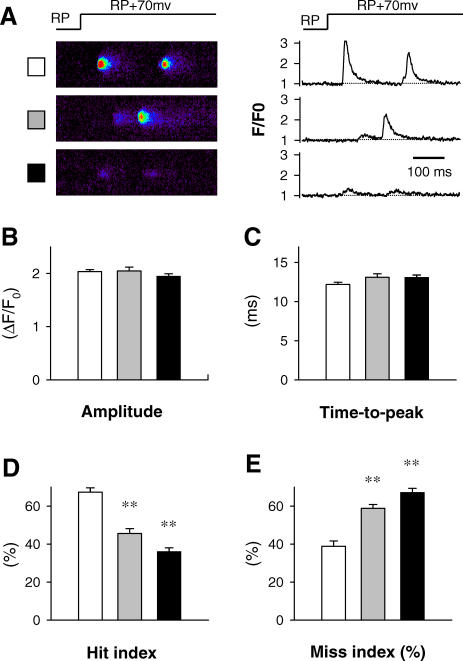
Triggering a Ca^2+^ Spark by Activating a Single LCC (A) Representative loose-patch confocal images (left) and their time profiles (right) show that a 70-mV depolarization from resting potential evoked RyR Ca^2+^ sparks (high-amplitude, rapid-takeoff events) and LCC Ca^2+^ sparklets (flat, slow events) in a stochastic manner. Images were selected from the control (top, showing two sparks that were triggered), CHT (middle, showing the first sparklet, with a 21-ms time to peak, that failed to trigger a spark), and DHT (bottom, showing two sparklets without triggering sparks) groups. (B) and (C) The amplitude (B) and the time to peak (C) of Ca^2+^ sparks did not differ among groups. Each datum was an average of more than 120 events from six or more animals. The bars represent the following groups: open bar, control; gray bar, CHT; and solid bar, DHT. (D) and (E) The “hit index” (D) represents the percentage of Ca^2+^ sparks that were apparently triggered by the first Ca^2+^ sparklets, whereas the “miss index” (E) denotes the percentage of depolarization pulses that did not trigger any Ca^2+^ sparks. The percentages were first calculated on a single-cell basis, and then averaged among more than 50 cells from six or more animals. The bars represent the following groups: open bar, control; gray bar, CHT; and solid bar, DHT.

To determine whether and how the intermolecular coupling between LCCs and RyRs is modified in different stages of hypertrophy, we utilized loose-patch confocal imaging [9] to visualize simultaneously the Ca^2+^ sparklet from an LCC and the triggered Ca^2+^ sparks from RyRs. The visibility of LCC sparklets was enhanced by adding to the pipette solution 20 mM Ca^2+^ and 10 μM FPL64176, an LCC agonist that decreases the channel opening frequency, but prompts long openings [9,18,19]. Upon depolarization of the on-cell membrane patch, confocal line-scan imaging detected two distinct populations of local Ca^2+^ signals (Figure 2A): the steep, high-amplitude events, sensitive to ryanodine, were Ca^2+^ sparks from RyRs; the flat, low-amplitude events, resistant to ryanodine, but sensitive to nifedipine, were Ca^2+^ sparklets from individual LCCs [9]. Examination of Ca^2+^ sparks identified no significant differences in spark amplitude (Figure 2B) and time to peak (Figure 2C), indicating that the spark generation process per se was basically unaltered during hypertrophy.

We then examined the probability of LCC-RyR coupling. The three recordings in Figure 2A, selected from the control, CHT, and DHT groups, show that individual LCC Ca^2+^ sparklets activated Ca^2+^ sparks in a hit-or-miss fashion. To quantify the probability for this stochastic process, we derived a “hit index” from the percentage of Ca^2+^ sparks that were apparently triggered by the first Ca^2+^ sparklets. The hit index revealed a significant decay from 67 ± 2% in the control to 46 ± 3% in CHT and 36 ± 2% in DHT (Figure 2D). As a backup of this result, we also parameterized a “miss index” by counting the percentage of depolarization pulses that occurred without triggering any Ca^2+^ sparks. The “bare” traces increased from 39 ± 3% in the control to 59 ± 2% in CHT and 67±2% in DHT (Figure 2E). Hit index and miss index, based on independent statistics from the loose-patch imaging data, showed congruously that the intermolecular coupling efficiency between LCCs and RyRs decreased significantly not only in DHT, but also in CHT.

**Figure 3:**
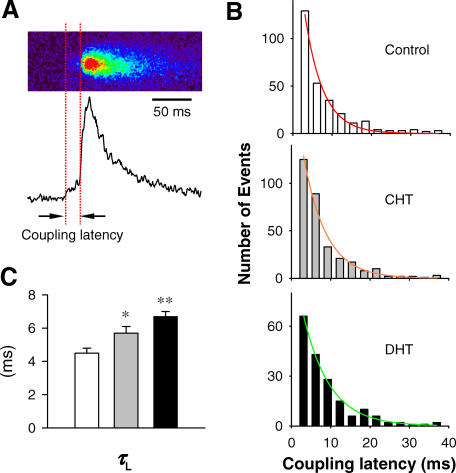
Kinetics of LCC-RyR Coupling (A) Illustration of a typical Ca^2+^ spark image (top) and its time profile (bottom) showing the coupling latency from the onset of an LCC Ca^2+^ sparklet to the takeoff of a triggered RyR Ca^2+^ spark. (B) Distribution (bars) and exponential fits (lines) of coupling latency in the control (top), CHT (middle), and DHT (bottom) groups. (C) Time constants of coupling latency (*τ*_L_) determined by the fits in (B). The values of error bars were given by Sigmaplot software (http://www.systat.com/products/sigmaplot/) during fitting. The bars represent the following groups: open bar, control; gray bar, CHT; and solid bar, DHT. A single asterisk (*) indicates *p* < 0.05, and double asterisks (**) indicate *p* < 0.01 compared with the control.

To further quantify the intermolecular aspects of E-C coupling during hypertrophy, we went on to measure the kinetics of LCC-RyR coupling. The LCC-RyR coupling latency was gauged as the delay from the onset of an LCC sparklet to the takeoff of a triggered RyR spark (Figure 3A). In each experimental group, the coupling latency had a monotonically decaying distribution (Figure 3B). Exponential fitting showed that the time constant for LCC-RyR coupling increased progressively from 4.5 ± 0.3 ms to 5.7 ± 0.4 ms in CHT and to 6.7 ± 0.3 ms in DHT (Figure 3C).

With the prolongation of LCC-RyR coupling latency, an LCC Ca^2+^ sparklet with a limited lifetime would have a decreased chance to activate RyR Ca^2+^ release. Therefore, the slowed coupling kinetics agrees well with the decreased chance of hit or increased chance of miss of LCC-RyR coupling. Taken together, our data provide unequivocal evidence that LCC-RyR coupling undergoes a progressive failure during hypertrophy. Notably, this modification occurs not only in DHT, but also in CHT, indicating that the intermolecular failure starts as early as the compensated stage, when the cellular E-C coupling appears intact.

### Cellular Tolerance against Intermolecular Failure

**Figure 4:**
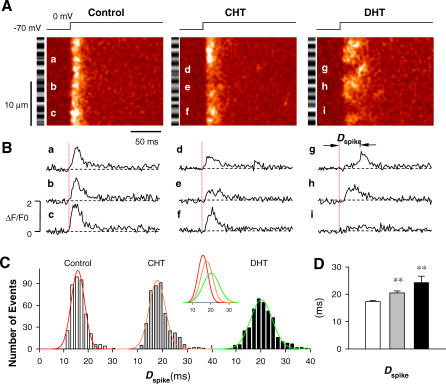
Visualization of “Ca^2+^ Spikes” at Individual T-tubule-SR Junctions (A) Representative images in the control (left), CHT (middle), and DHT (right) groups. The cells were depolarized from −70 to 0 mV when 0.25 mM fluo-4 and 10 mM EGTA were included in the pipette electrode solution. The black-and-white strip next to each image is a positioning reference of Z-lines and T-tubules derived from the background fluo-4 fluorescence prior to depolarization. The lowercase letters, a–i, indicate the positions of Ca^2+^ spikes whose time courses are shown in (B). (B) Typical time courses of Ca^2+^ spikes at positions noted in (A). (C) Distributions and their Gaussian fits of the time to peak of Ca^2+^ spikes (*D*_spike_) in the control (open bars and red line), CHT (gray bars and orange line), and DHT (solid bars and green line) groups. The measurement of *D*_spike_ is illustrated in trace g in (B). The inset contrasts the fitted distributions among groups. (D) Comparison of the average of *D*_spike_ among groups. Each datum represents the average of approximately 400 spikes in seven or more cells from three or more animals. The bars represent the following groups: open bar, control; gray bar, CHT; and solid bar, DHT. A single asterisk (*) indicates *p* < 0.05 compared with the control.

How then, if at all, is LCC-RyR intermolecular failure logically linked to the global performance of E-C coupling? To seek an answer, we examined the cell-wide patterning of Ca^2+^ release activities of local Ca^2+^ release sites. We performed whole-cell voltage-clamp experiments and dialyzed into the cell 10 mM EGTA, a slow Ca^2+^ buffer that suppresses the spatiotemporal integral of Ca^2+^ without interfering with CICR [19,20]. When the cell membrane was depolarized to 0 mV, cell-wide Ca^2+^ release was visualized as a line of discrete “Ca^2+^ spikes” [19,20] (Figure 4A), each occurring at a T-tubule–SR junction (identified by the bright Z-line structure in the black-and-white strips next to the images in Figure 4A). Notably, the Ca^2+^ spikes tended to be desynchronized in the groups with hypertrophy. To quantify this phenomenon, we measured the time delay from depolarization to the peak of each Ca^2+^ spike (*D*_spike_, illustrated in trace g of Figure 4B). We found that the time distributions of *D*_spike_ became dispersed, with the peaks shifting rightward in CHT and DHT in a progressive manner (Figure 4C). Accordingly, the average *D*_spike_ increased significantly in CHT and DHT (Figure 4D). These results, consistent with the loose-patch data, provided independent evidence that a microscopic defect of E-C coupling indeed occurs as early as CHT.

**Figure 5:**
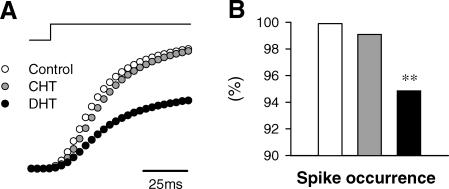
Global Performance of Ca^2+^ Spikes (A) Comparison of the time integral of Ca^2+^ spikes in the control (open circle), CHT (gray circle), and DHT (solid circle) groups. Each trace was an average of seven or more cells from three or more animals. (B) Rate of Ca^2+^ spike occurrence at individual T-tubules during depolarization to 0 mV. The bars represent the following groups: open bar, control; gray bar, CHT; and solid bar, DHT. The double asterisks (**) indicate *p* < 0.01 compared with the control.

The unique advantage of Ca^2+^ spike imaging is that it links the local behavior of Ca^2+^ release to the global performance of E-C coupling. Despite the desynchronization of Ca^2+^ spikes, we found that the cell-wide time integral of Ca^2+^ spikes in CHT could still reach a level similar to the control (Figure 5A). In accordance, Ca^2+^ spikes could still be activated at nearly 100% of T-tubules (Figure 5B). This phenomenon suggested that within a certain limit, for which we adopted the cybernetic term “stability margin,” the amount of cell-wide Ca^2+^ release is stabilized against a moderate variation of LCC-RyR intermolecular coupling efficiency. However, when the failure of LCC-RyR coupling steps beyond the stability margin, global performance of E-C coupling falls due to insufficient activation of Ca^2+^ release units (CRUs) (Figure 5B) and a degraded time integral of Ca^2+^ release (Figure 5A).

**Figure 6:**
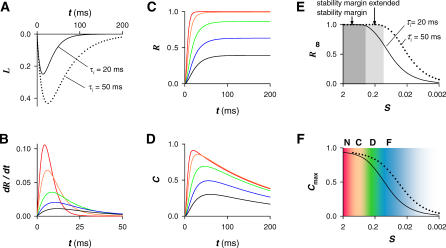
Simulation of Relationship between E-C Coupling Performance and LCC-RyR Intermolecular Coupling Efficiency (A) Time course of *I*_Ca_ used in the numerical simulation. Curves were calculated as the product of exponential activation (time constant *τ* = 20 ms) and inactivation (time constant *τ*_i_ = 20 and 50 ms for the solid and dashed lines, respectively). (B–D) Simulated time profile of the CRU activation per unit time *(dR/dt)* (B), cumulative CRU activation *(R)* (C), and the Ca^2+^ transient (D) when the RyR sensitivity coefficient *S* was set at 1 (red), 0.5 (orange), 0.2 (green), 0.1 (blue), or 0.05 (black). (E) Relationship between the maximal accumulative CRU activation (*R*_∞_) and *S*. Note the extension of the stability margin (from dark gray to light gray) when *τ*_i_ was prolonged from 20 ms (solid line) to 50 ms (dashed line). (F) The *S* dependence of peak amplitude of the Ca^2+^ transient (*C*_max_) when *τ*_i_ = 20 ms (solid line) or 50 ms (dashed line). The letters on the top denote the stages that mimic prominent E-C coupling characteristics of the control (N), CHT (C), DHT (D), and heart failure (F) groups.

As a proof of principle, we simulated the dependence of the fractional activation of CRUs *(R)* on the sensitivity *(S)* of RyRs to LCC Ca^2+^ influx *(L)*. Based on the stochasticity of LCC-RyR coupling [9], the *R* per unit time *(dR/dt)* relates positively to *L, S,* and RyR availability. Due to the refractoriness of Ca^2+^ release [19,21], the RyR availability is proportional to (1 − *R*). Therefore, by the first order of approximation,

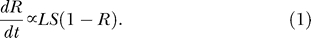
The triggered Ca^2+^ release then produces a Ca^2+^ transient *C(t),* which can be described by

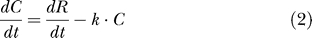
where *k* represents the apparent rate constant for Ca^2+^ removal from the cytosol. Assuming that both activation and inactivation of LCCs follow exponential kinetics (Figure 6A), Equation (1) predicts that, with the decrease of *S, dR/dt* becomes reduced and dispersed (Figure 6B), reproducing the data of *D*_spike_ in Figure 4C and 4D. Notably, despite the progressive decrease in *dR/dt,* both *R* and *C* were initially insensitive to *S* (red and orange lines in Figure 6C and 6D) until the stability margin reached its edge (Figure 6E and 6F), agreeing well with the data in Figure 5. Therefore, both numerical simulation and experimental data support the idea that a stability margin must be broached for global E-C uncoupling to occur.

To test the above principle directly, we further designed an experiment (Figure S2A) utilizing the principle that the LCC-RyR coupling efficiency depends on the square of unitary Ca^2+^ current [22]. When LCC unitary current was linearly modulated by membrane voltage [9], the decrease of LCC-RyR coupling efficiency resulted in a sigmoid relationship between membrane voltage and Ca^2+^ transient amplitude (Figure S2B), with the stability margin (light-colored bands in Figure S2B) agreeing well with the simulated result (gray bands in Figure 6F). Furthermore, the dynamic range of the stability margin appeared as a function of the dwell time *(T)* of *I*_Ca_, which could also be reproduced by the simulation in which slowed *I*_Ca_ inactivation (dashed line in Figure 6A) resulted in an extended stability margin (dashed lines in Figure 6E and 6F).

### Seeking Potential Mechanisms underlying the Intermolecular Failure

In the present study, our results suggested that the intermolecular failure started as early as the CHT stage, when cellular E-C coupling still appeared intact. It was therefore intriguing to explore the mechanism underlying the intermolecular dysfunction. Many studies have shown that SERCA activity is down-regulated, and Na/Ca exchange up-regulated, in failing heart cells, both preventing the SR from filling [8,11]. In order to test whether SR Ca^2+^ load plays a role in the intermolecular coupling defect, we measured the caffeine-induced Ca^2+^ transients using a lower-affinity Ca^2+^ indicator, fluo-5F, to avoid indicator saturation [23]. However, as previously reported for other hypertrophy models [13,24], we could not identify a difference in SR Ca^2+^ load in resting cells of the control, CHT, and DHT groups (Figure S3).

**Figure 7:**
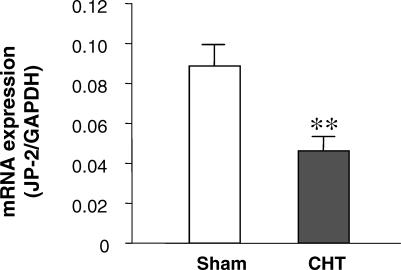
Expression of Junctophilin-2 (JP-2) in Ventricular Myocytes of Sham-Operated and CHT Rats RNA of JP-2 was quantified by real-time quantitative RT-PCR; the expression of JP-2 relative to GAPDH was found to be significantly lower in the control (0.089 ± 0.010) compared to CHT (0.046 ± 0.007) group (*n* = 4 rats). Double asterisks (**) indicate *p* < 0.01 compared with control.

It has been postulated that a structural rearrangement between SR and cell/T-tubule membrane may underlie the defective E-C coupling in failing heart cells [13]. Many laboratories found that the T-tubule structure indeed undergoes structural degradation during heart failure [25,26], leaving RyRs orphaned [27]. However, when we used the same method [27] to examine LCC-RyR co-localization (Figure S4A), we could not detect a change in our hypertrophy models (Figure S4B), indicating that the membrane restructuring observed in the late, failing stage could not be detected in the early, hypertrophy stage. To explore whether any molecular event related to structural uncoupling accompanies the onset of intermolecular failure, we examined in the CHT group the expression of junctophilin-2 (JP-2), a protein anchoring SR to cell/T-tubule membrane and being down-regulated in mouse models of dilated cardiomyopathy [28]. Quantitative analysis using real-time reverse transcriptase PCR (RT-PCR) showed that JP-2 mRNA expression, normalized by GAPDH, was significantly decreased in CHT (Figure 7). Therefore, despite the absence of an apparent structural modification, a subtle physical alteration associated with decreased JP-2 expression is expected to contribute to the earliest change in LCC-RyR coupling efficiency.

## Discussion

The present study aimed to establish the intermolecular basis for the modified E-C coupling during hypertrophy that eventually leads to heart failure. Using rat models of CHT and DHT, we characterized the cardiac E-C coupling at the cellular, sub-cellular, and intermolecular levels. For DHT, our data provided the molecular interpretation that the slowed response of RyR to unitary LCC triggers results in an increased chance of failure in LCC-RyR coupling, which then leads to a degradation in intracellular Ca^2+^ transients. For CHT, we found unexpectedly that intermolecular failure proceeds in the background without altering cellular function of E-C coupling. The latter is of prominent clinical significance, as it demonstrated a hint of pathogenesis at a very early stage of heart failure.

### Direct Determinants of E-C Coupling

The development of hypertrophy and heart failure is a progressive remodeling characterized by increased wall thickness, impaired diastolic function, excessive fibrosis, increased myocardial stiffness, and altered expression of genes encoding contractile, regulatory, and structural proteins [1–4]. The present study has been focused on the mechanisms that modify Ca^2+^ transient generation, which depends directly on the following factors:

LCC activity: Depending on different models, LCC activity is found to be either increased, decreased, or unchanged during the transition from hypertrophy to heart failure [13,14]. Our data in Figure 1E and 1G support the idea that LCC regulation does not play a major role in the modification of E-C coupling during hypertrophy.

Ca^2+^ release from RyRs: Depolarization-induced Ca^2+^ transients can be explained by the spatiotemporal summation of Ca^2+^ sparks [29,30]. Spontaneous Ca^2+^ sparks with increased amplitude were found in hypertrophied spontaneously hypertensive rats [24], but not in salt-sensitive rats [13]. In the present model of hypertrophy, Ca^2+^ sparks were triggered by LCC Ca^2+^ influx. We identified no difference in Ca^2+^ spark parameters, but found a desynchronization of Ca^2+^ spike patterning. Desynchronized Ca^2+^ release has been found previously in heart failure [31,32], and attributed in part to an absence of early repolarization in the action potential [32]. Here we show that desynchronization of Ca^2+^ release has already begun in CHT, and is induced by decreased efficiency of LCC-RyR coupling.

Efficiency of LCC-RyR coupling: The degraded E-C coupling gain with unchanged *I*_Ca_ and Ca^2+^ sparks lead to the idea that the efficiency of the LCC current to activate RyR Ca^2+^ release may be decreased [13]. Here, using a loose-patch spark imaging technique, we provided direct evidence that the coupling kinetics between a single LCC and RyRs is slowed during hypertrophy, resulting in a status of intermolecular failure. Moreover, we demonstrated that the deficiency occurs as early as CHT when the cellular performance of E-C coupling appears normal.

Based on the above discussion, we believe that decreased LCC-RyR coupling efficiency is a major problem of E-C coupling in hypertrophy, and also may represent the earliest functional hint that heart failure is developing.

### Factors underlying LCC-RyR Coupling Efficiency

To understand the modification of LCC-RyR coupling efficiency, the following aspects need to be considered:

SR lumenal regulation: SR Ca^2+^ load not only determines the amount of releasable Ca^2+^, but also modulates the RyR sensitivity to Ca^2+^ [8]. In many heart failure models, decreased SR Ca^2+^ load [33,34] due to reduced expression of SERCA protein [11,33] or impaired SERCA regulation by phospholamban [35,36] are responsible, at least in part, for the E-C uncoupling. Improving SR Ca^2+^ uptake function by expression of SERCA or the pseudophosphorylated mutant of phospholamban can indeed improve the cardiac performance in heart failure models [37,38]. Even though, in many failing models [13,24,39], including the hypertrophy models in the present study (Figure S3), resting cardiomyocytes with a normal SR Ca^2+^ load still exhibit degraded efficiency for LCC Ca^2+^ influx to activate Ca^2+^ transients, suggesting that an SR load-independent mechanism also underlies the defective E-C coupling.

Molecular Modulation of RyRs: In failing hearts, it has been found that RyRs are hyperphosphorylated, and the resulting dissociation of FKBP12.6 is a major cause of the defective E-C coupling [10,40]. Recently, contrary evidence has also been presented [41]. Therefore, whether the defective LCC-RyR coupling involves RyR modification is still under debate.

Structural coupling with LCCs: It has been postulated [42], and recently simulated [43], that the local Ca^2+^ concentration generated by LCCs and “seen” by RyRs varies by orders of magnitude with the distance between junctional SR and surface/T-tubule membrane. Hence, even a subtle change in junctional structure may severely alter LCC-RyR signaling efficiency. In heart failure models, structural degradation is found in the T-tubule system [25,26], leaving many RyRs orphaned [27]. Associated with the structural change, JP-2, a protein that anchors junctional SR to cell membrane, is down-regulated in cardiomyopathy models showing decreased Ca^2+^ transients but normal *I*_Ca_ [28]. In the present study, we examined an earlier stage of hypertrophy exhibiting normal Ca^2+^ transients and contractility. Although a structural change was not yet detectable, we did see that JP-2 expression was curtailed by half.

Taken together, LCC-RyR coupling efficiency is a multifactorial issue. Presently, the structural/ultrastructural modification associated with decreased JP-2 expression, which would physically uncouple LCCs and RyRs, is the most plausible mechanism underlying the functional coupling failure between LCCs and RyRs. Therefore, the upstream pathways that regulate JP-2 may become putative candidates for therapeutic targeting against heart failure.

### Relationship between Global Performance and Intermolecular Failure

An interesting finding in the present study is that intermolecular failure of LCC-RyR signaling occurs as early as CHT when global E-C coupling appears intact. This finding is backed up by several independent lines of evidence, including (1) decreased chance of hit, (2) increased chance of miss, (3) the prolonged latency of LCC-RyR coupling, and (4) the prolonged delay and progressive temporal dispersion of cell-wide Ca^2+^ spikes. An intriguing question is why and to what extent cellular E-C coupling is capable of tolerating intermolecular failure.

In heart cells, the intermolecular coupling between LCCs and RyRs is intrinsically stochastic, rather than deterministic [9]. The beauty of such a mode of control is that a RyR CRU that once fails to respond to an LCC opening still has a similar chance to be activated by later LCC openings [9]. In the present study, we demonstrated that slowed kinetics and increased chance of miss in LCC-RyR coupling in CHT did not alter the time integral of Ca^2+^ spikes (Figure 5A), suggesting that even when the CRUs are less responsive, they are sufficiently activated before *I*_Ca_ inactivation at the expense of Ca^2+^ release desynchronization. This speculation is fully supported by our numerical simulation. With the decrease of *S,* although the *dR/dt* (CRU activation per unit time) becomes lower in peak, it lasts longer in time (Figure 6B). Within the stability margin, these two trends compensate for each other, keeping the cumulative CRU activation *(R)* relatively unchanged. However, with further decrease of LCC-RyR coupling efficiency, an increasing fraction of CRUs tends not to be activated before *I*_Ca_ inactivation (Figure 5B; green, blue, and black lines in Figure 6C); global performance of Ca^2+^ release declines.

In testing the principle that a stability margin must be broached for cellular uncoupling to occur, we found that decreasing LCC-RyR coupling efficiency by a factor other than hypertrophy obeyed the same principle. Therefore, the stability margin principle has general meaning in linking the intermolecular aspect and the global aspect of E-C coupling. In addition, both experimental and numerical data support the idea that prolonged *I*_Ca_ exposure can extend the stability margin (Figure 6E and Figure S2B). With an extended stability margin, the global performances become less influenced by intermolecular failure. Therefore, finding strategies that extend the stability margin, e.g., by targeting LCC inactivation, may have potential implications in developing therapeutic treatments against heart failure.

## Materials and Methods

### Aortic stenosis model.

Ascending aortic stenosis surgery was performed in male Sprague-Dawley rats (body weight, 50–55 g, obtained from the Medical Experimental Animal Center of Peking University) as described previously [17]. Briefly, rats were anesthetized with a ketamine-xylazine mixture (5:3, 1.32 mg/kg intraperitoneally). The thorax was opened and a silver clip (0.9-mm inside diameter) was placed on the ascending aorta. Sham-operated animals underwent an identical procedure but without the clip. To characterize the model, echocardiographic measurements were made using a Vivid 7 Dimension cardiovascular ultrasound system (GE Healthcare, Fairfield, Connecticut, United States) as described previously [43]. A 2.0-F high-fidelity nanometer-tipped catheter (SPR-838; Millar Instruments, Houston, Texas, United States) was introduced through the right carotid artery and retrogradely across the aortic valve in the left ventricle to measure hemodynamic parameters [43]. The parameters measured are listed in Table S1.

### Whole-cell patch clamp.

Single ventricular myocytes were isolated using an enzymatic method described previously [44]. The cells were bathed in an extracellular solution containing (in mM): 137 NaCl, 4.0 KCl, 1.0 CaCl_2_, 1.2 MgCl_2_, 1.2 NaH_2_PO_4_, 10 glucose, 0.02 tetrodotoxin, and 10 HEPES, (pH 7.35) adjusted with NaOH. An EPC7 amplifier (List Medical Electronic, Darmstadt, Germany) was used for the whole-cell patch clamp technique. For Ca^2+^ transient measurement, the patch pipette filling solution contained (in mM): 127 CsCl, 10 NaCl, 1 MgCl_2_, 5 MgATP, 15 tetraethylammonium chloride, 10 HEPES, and 0.2 fluo-4 pentapotassium (Molecular Probes, Eugene, Oregon, United States), (pH 7.2) adjusted with CsOH. When Ca^2+^ spikes occurring at T-tubules were measured, 10 mM EGTA and 4 mM CaCl_2_ were included in the pipette solution with the CsCl decreased to 115 mM. *I*_Ca_ was activated by 200-ms depolarization pulses at 10-s intervals.

### Loose-seal patch clamp.

The loose-seal patch clamp technique, as described previously [9], was performed using the same setup as in the whole-cell patch clamp. A glass pipette with a resistance (R_p_) of 3–5 MΩ (∼1 μm at the tip) was gently pressed onto the cell surface to form a low-resistance seal (R_s_ = 20–30 M). The patch membrane voltage (V_P_) was determined based on resting potential (RP) and command voltage (V_com_) by V_P_ = RP − V_com_R_s_/(R_s_+R_p_). The pipettes were filled with extracellular solution except that 20 mM Ca^2+^ and 10 μM FPL64176 were included to facilitate the visualization of Ca^2+^ sparklets from LCCs.

### Confocal imaging.

For whole-cell experiments, the Ca^2+^ indicator fluo-4 was already in the pipette solution. To load fluo-4 in loose-patch experiments, cells were incubated in 2.5 μM fluo-4 AM (Molecular Probes) in extracellular solution for about 5 min in the dark at 37 °C. Ca^2+^ transients, Ca^2+^ spikes from individual T-tubules, and Ca^2+^ sparks were measured using a Zeiss LSM-510 inverted confocal microscope (Carl Zeiss, Oberkochen, Germany). All image data were taken in the line-scanning mode along the long axis of the myocyte excited at 488 nm. The Ca^2+^ level was reported as *F/F*_0_, where *F*_0_ is the resting or diastolic fluo-4 fluorescence.

### RNA isolation and quantitative real-time RT-PCR.

Total RNA from isolated ventricular myocytes was extracted using Trizol reagent, and first-strand cDNA was generated using the ImProm-II Transcription System (Promega, Madison, Wisconsin, United States). Quantitative real-time RT-PCR was performed using the primers of junctophilin type-2 (5′-AGGCGGGTGCCAAGAAGAAG-3′; 5′-CGATGTTCAGCAGGATCACCA-3′) and GAPDH (5′-ATCAAGAAGGTGGTGAAGCA-3′; 5′-AAGGTGGAAGAATGGGAGTTG −3′). Amplifications were performed in 35 cycles using an Opticon continuous fluorescence detection system (Bio-Rad, Hercules, California, United States) with SYBR Green fluorescence (Molecular Probes). Each cycle consisted of 30 s at 94 °C, 30 s at 56 °C, and 30 s at 72 °C. All samples were quantified using the comparative Ct method for relative quantitation of gene expression, normalized to GAPDH [45].

### Statistical analysis.

Results are expressed as mean plus or minus the standard error of the mean (SEM). Statistical analysis was performed using the Student *t*-test for unpaired samples; a value of *p* < 0.05 was considered significant. When fitted data were compared, their difference was divided by the standard error and compared with the standard normal distribution. During the experiments, we considered the time difference between CHT (7–9 wk after surgery) and DHT (9–11 wk). Sham-operated controls were set for each group. As the data from these two control groups did not differ significantly, they were combined together as the “control” group in this manuscript.

## Supporting Information

Figure S1 Comparison of Cell Capacitance, Contraction Amplitude, and Kinetics of I_Ca_ among Control, CHT, and DHT Comparison of cell capacitance (A), contraction amplitude (B), and kinetics of *I*_Ca_ (C–E) among control, CHT, and DHT groups (*n* ≥ 16 cells from four or more rats for each group). The kinetics of *I*_Ca_ is characterized by the fast (C) and slow (D) time constants. A single asterisk (*) indicates *p* < 0.05.
(19 KB PDF)

Figure S2 Experimental Test of the Numerical Model in Figure 6
(A) Representative records of the experiment. The cell membrane was first depolarized beyond reversal potential (to +80 mV), which could not activate Ca^2+^ transient, and then repolarized to various potentials (−20 to +50 mV), which activated *I*_Ca_ with different amplitudes. The resulted Ca^2+^ transient was recorded by confocal microscopy.
(B) The sigmoid relationship between Ca^2+^ transient amplitude and membrane potential. Note the leftward shift of the curves when *I*_Ca_ duration shortens.
(64 KB PDF)

Figure S3 SR Ca^2+^ Load as Assessed by the Amplitude of Ca^2+^ Transient Induced by Quick Local Perfusion of 20 mM Caffeine The low-affinity Ca^2+^ indicator, fluo-5F was used to avoid indicator saturation. No significant difference (*p* > 0.05) could be identified among the control, CHT, and DHT groups (*n* ≥ 15 cells from three or more rats for each group).
(17 KB PDF)

Figure S4 LCC Distribution Relative to RyRs (A) Representative immunostaining images of LCC (red) and RyR (green) distribution in cardiomyocytes.
(B) Percentage of LCC fluorescence co-localized with that of RyRs (*n* ≥ 10 cells from three rats in each group).
(31 KB PDF)

Table S1 In Vivo Hemodynamic and Echocardiographic Measurements (73 KB PDF)

## Abbreviations

CHT: compensated hypertrophy
CICR: Ca^2+^-induced Ca^2+^ release
CRU: Ca^2+^ release unit
DHT: decompensated hypertrophy
E-C coupling: excitation-contraction coupling
*I*_Ca_ L-type Ca^2+^: channel current
LCC: L-type Ca^2+^ channel
RT-PCR: reverse transcriptase PCR
RyR: ryanodine receptor
SERCA: sarcoplasmic reticulum Ca^2+^ pumps
SR: sarcoplasmic reticulum
