# Supplementary material for: Intermolecular Failure of L-type Ca2+ Channel and Ryanodine Receptor Signaling in Hypertrophy
Source: PLoS Biol. 2007 Jan 9;5(2):e21. doi: 10.1371/journal.pbio.0050021 (PMC1764437; doi:10.1371/journal.pbio.0050021)
Supplement: Supplementary file 1 [file pbio.0050021.sg001.pdf]

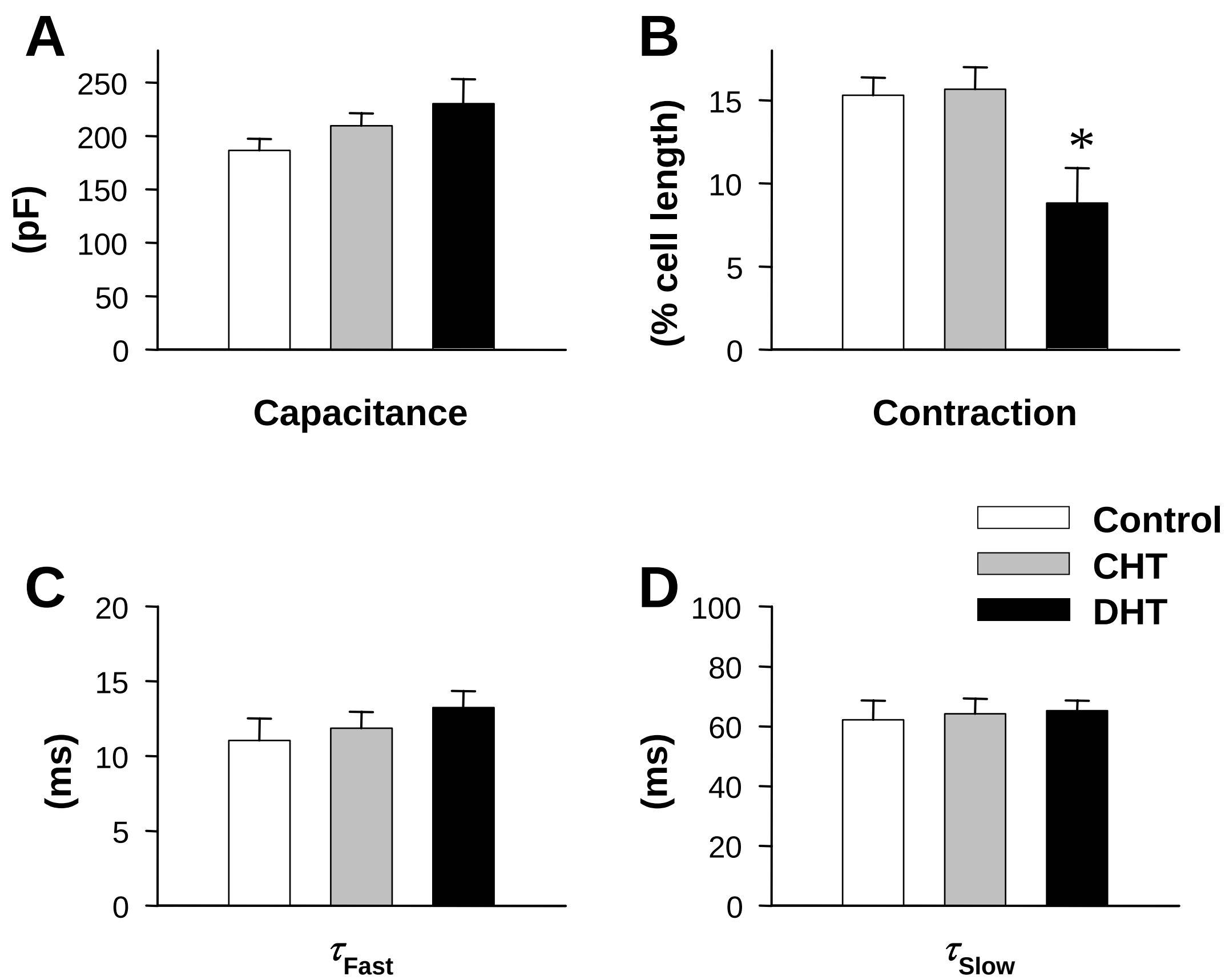

A
250
200
150
100
50
0
(pF)
Capacitance
B
15
10
5
0
(% cell length)
*
Contraction
Control
CHT
DHT
C
20
15
10
5
0
(ms)
τFast
D
100
80
60
40
20
0
(ms)
τSlow
