# Supplementary material for: Intermolecular Failure of L-type Ca2+ Channel and Ryanodine Receptor Signaling in Hypertrophy
Source: PLoS Biol. 2007 Jan 9;5(2):e21. doi: 10.1371/journal.pbio.0050021 (PMC1764437; doi:10.1371/journal.pbio.0050021)
Supplement: Supplementary file 2 [file pbio.0050021.sg002.pdf]

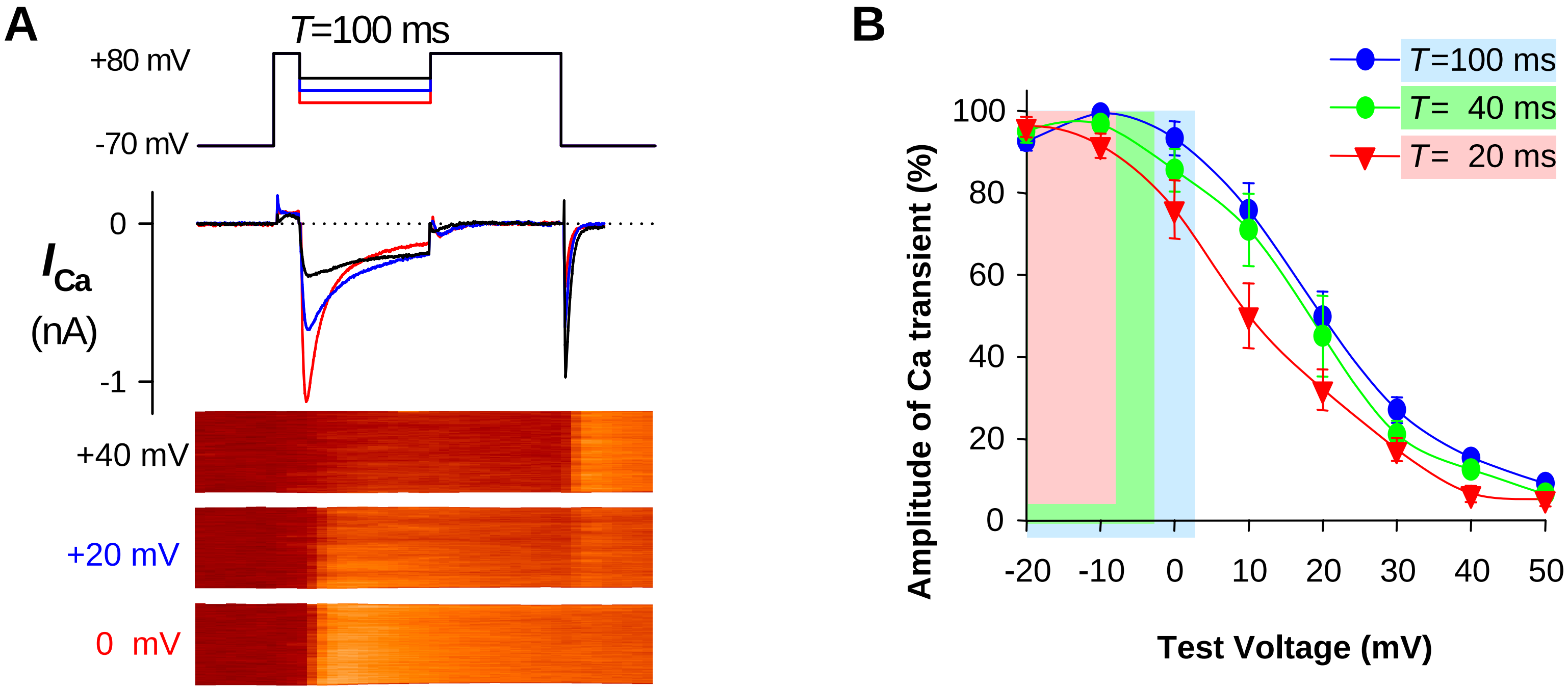

A
T=100 ms
+80 mV
-70 mV
0
-1
I Ca
(nA)
+40 mV
+20 mV
0 mV
B
T=100 ms
T= 40 ms
T= 20 ms
Amplitude of Ca transient (%)
100
80
60
40
20
0
-20
-10
0
10
20
30
40
50
Test Voltage (mV)
