# Supplementary material for: Intermolecular Failure of L-type Ca2+ Channel and Ryanodine Receptor Signaling in Hypertrophy
Source: PLoS Biol. 2007 Jan 9;5(2):e21. doi: 10.1371/journal.pbio.0050021 (PMC1764437; doi:10.1371/journal.pbio.0050021)
Supplement: Supplementary file 3 [file pbio.0050021.sg003.pdf]

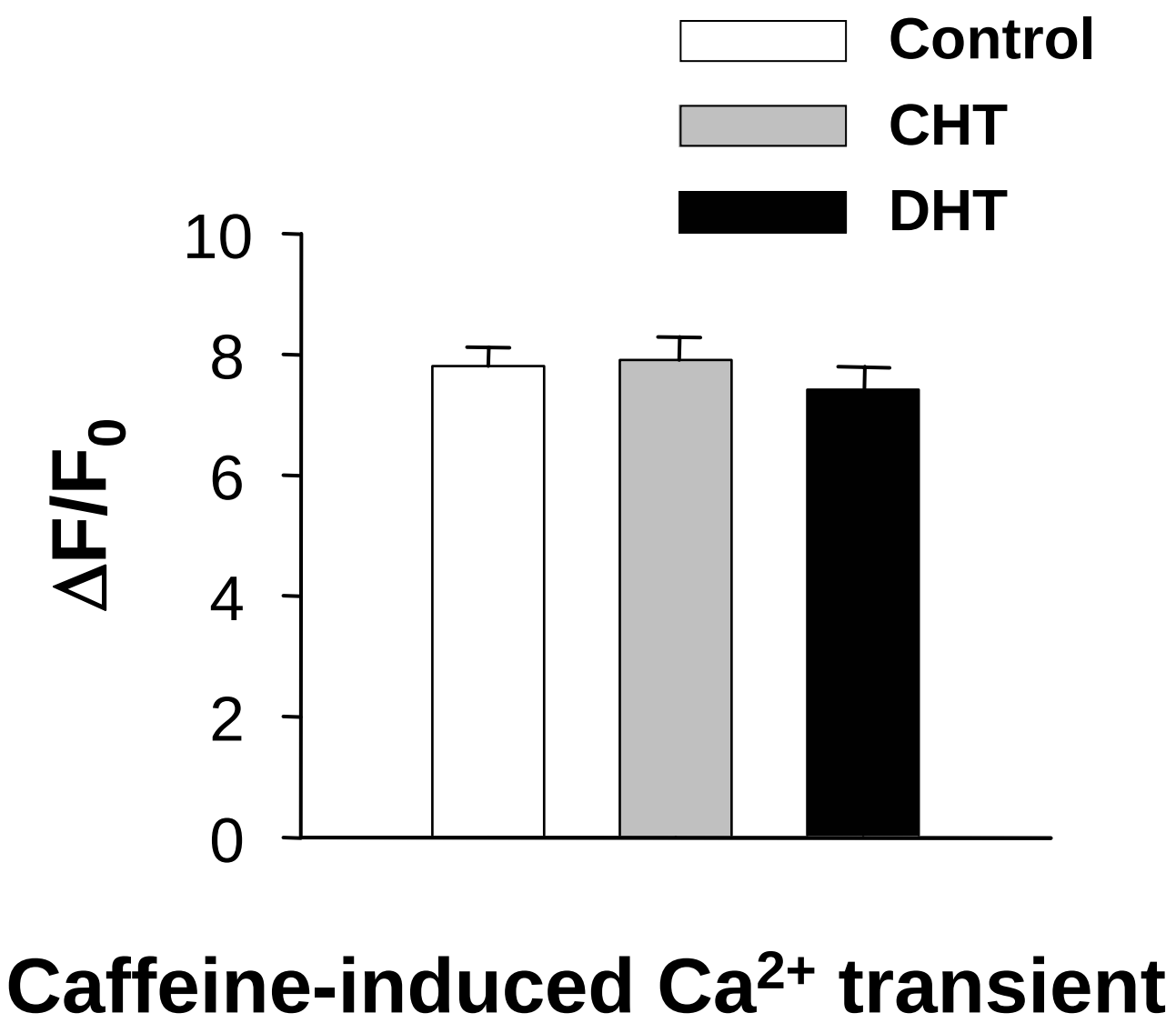
Control
CHT
DHT
10
8
6
4
2
0
ΔF/F0
Caffeine-induced Ca2+ transient
