# Supplementary material for: Intermolecular Failure of L-type Ca2+ Channel and Ryanodine Receptor Signaling in Hypertrophy
Source: PLoS Biol. 2007 Jan 9;5(2):e21. doi: 10.1371/journal.pbio.0050021 (PMC1764437; doi:10.1371/journal.pbio.0050021)
Supplement: Supplementary file 4 [file pbio.0050021.sg004.pdf]

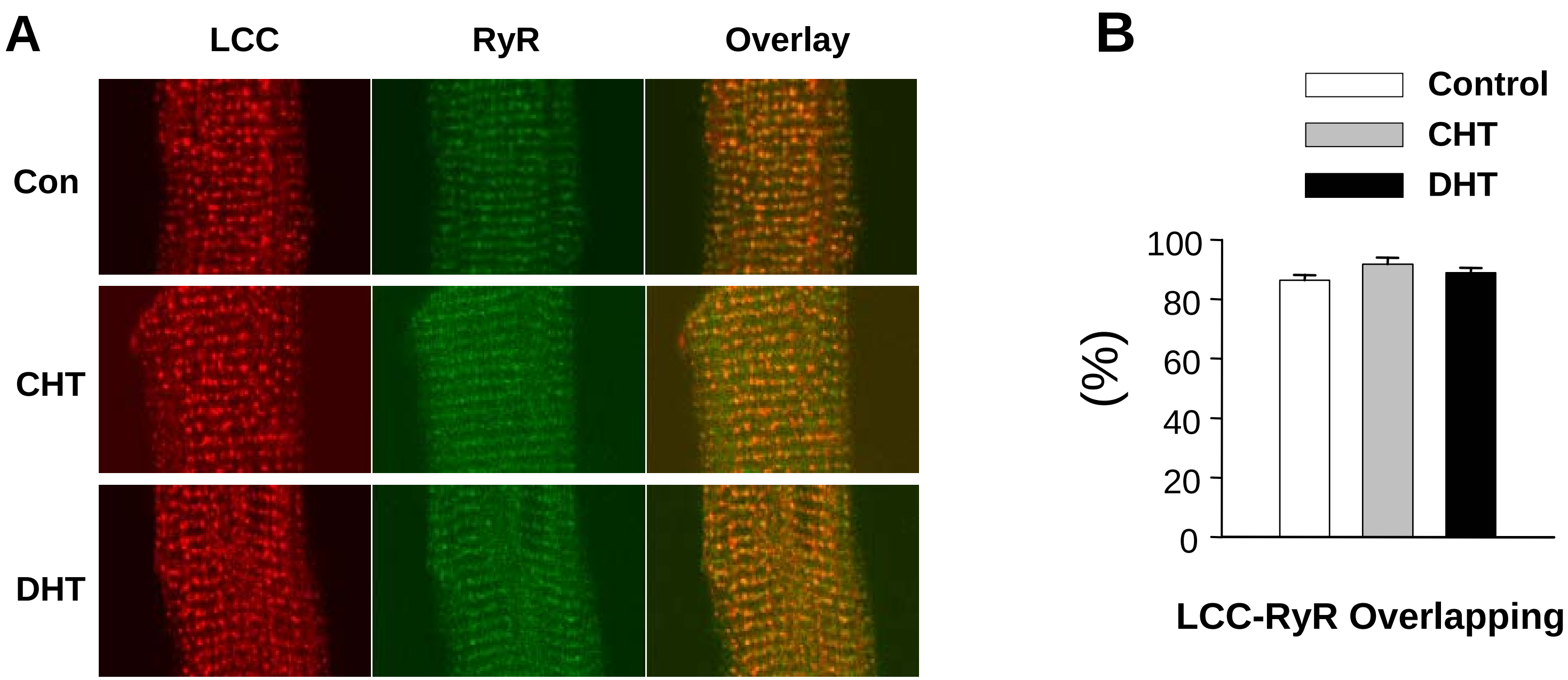
A
LCC
RyR
Overlay
Con
CHT
DHT
B
Control
CHT
DHT
100
80
60
40
20
0
(%)
LCC-RyR Overlapping
