# Supplementary material for: Intermolecular Failure of L-type Ca2+ Channel and Ryanodine Receptor Signaling in Hypertrophy
Source: PLoS Biol. 2007 Jan 9;5(2):e21. doi: 10.1371/journal.pbio.0050021 (PMC1764437; doi:10.1371/journal.pbio.0050021)
Supplement: Supplementary file 5 [file pbio.0050021.st001.pdf]

Xu *et al*,

Suppl.Table 1. *In Vivo* Hemodynamic and Echocardiographic Measurements

	Con (n=8)	CHT (n=7)	DHT (n=7)
HR (beats/min)	348 ± 17	326 ± 23	345 ± 21
BW (g)	424 ± 9	401 ± 14	436 ± 23
LV mass (g)	0.97 ± 0.03	1.35 ± 0.07*	1.46 ± 0.078*
LV mass/BW (g/kg)	2.29 ± 0.07	3.4 ± 0.26*	3.4 ± 0.25*
LVD_d (mm)	7.15 ± 0.21	6.83 ± 0.14	7.9 ± 0.24*
LVD_s (mm)	3.6 ± 0.18	3.13 ± 0.14	5.26 ± 0.16*
FS%	50 ± 2	56 ± 2*	33 ± 2*
AW_d (mm)	2.06 ± 0.10	2.93 ± 0.21*	2.86 ± 0.22*
PW_d (mm)	2.05 ± 0.09	2.94 ± 0.21*	2.94 ± 0.14*
RWT	0.58 ± 0.03	0.86 ± 0.06*	0.75 ± 0.06*
LVSP (mm Hg)	106 ± 2	191 ± 4*	186 ± 5*
LVdevP/g (mm Hg/g)	100 ± 3	130 ± 7*	119 ± 7*
LV dP/dt_{max} (mm Hg/s)	7532 ± 228	7862 ± 251	7263 ± 115
LV dP/dt_{min} (mm Hg/s)	4867 ± 211	4987 ± 192	5248 ± 210
LVEDP (mm Hg)	5.1 ± 0.3	11.4 ± 0.4*	13.3 ± 0.5*

Con, Control; CHT, compensated hypertrophy; DHT, decompensated hypertrophy; HR, heart rate; BW, body weight; LV mass, left ventricular mass; LVD_d, left ventricular diastolic diameter; LVD_s, left ventricular systolic diameter; FS, fractional shortening; AW_d, anterior wall thickness; PW_d, posterior wall thickness; RWT, relative wall thickness calculated as the ratio of 2×posterioir wall thickness and LVD_d; LVSP, left ventricular systolic pressure; LVdevP/g, left ventricular developed pressure per g LV; LVEDP, left ventricular end diastolic pressure. *$P<0.05$ vs Control.
